# Poly-l-gamma-glutamic acid production by recombinant *Bacillus subtilis* without *pgsA* gene

**DOI:** 10.1186/s13568-018-0636-x

**Published:** 2018-07-03

**Authors:** Kazuhisa Sawada, Hiroyuki Araki, Yasushi Takimura, Kenta Masuda, Yasushi Kageyama, Katsuya Ozaki, Hiroshi Hagihara

**Affiliations:** 1 Global R&D-Biological Science Research, Kao Corporation, 2606 Akabane, Ichikai, Haga, Tochigi 321-3497 Japan; 2 Global R&D-Safety Science Research, Kao Corporation, 2606 Akabane, Ichikai, Haga, Tochigi 321-3497 Japan; 3 Global R&D-Strategy and Planning, Kao Corporation, 2-1-3 Bunnka, Sumida, Tokyo 131-8501 Japan

**Keywords:** Poly-gamma-glutamic acid, *Bacillus subtilis*, PgsBCA complex, Recombinant strain, l-PGA

## Abstract

Poly-gamma-glutamic acid (PGA) is a promising bio-based polymer that shares many functions with poly (acrylic acid) and its derivatives. Thus, technologies for efficient production and molecular size control of PGA are required to expand the application of this useful biopolymer. In *Bacillus* strains, PGA is synthesized by the PgsBCA protein complex, which is encoded by the *pgsBCA* gene operon, otherwise is known as *ywsC* and *ywtAB* operons and/or *capBCA* operon. Hence, we investigated responsible components of the PgsBCA complex in *B. subtilis* for over-production of PGA. In particular, we constructed genomic *pgsBCA* gene-deletion mutants of *B*. *subtilis*. And also, we assembled high copy-number plasmids harboring σA-dependent promoter, leading to high-level expression of all combinations of *pgsBCA*, *pgsBC*, *pgsBA*, *pgsCA*, *pgsB*, *pgsC*, and/or *pgsA* genes. Subsequently, PGA production of the transformed *B*. *subtilis* mutant was determined in batch fermentation using medium supplemented with l-glutamate. PGA production by the transformants introduced with *pgsBC* genes (lacking the genomic *pgsBCA* genes) was 26.0 ± 3.0 g L^−1^, and the enantiomeric ratio of d- and l-glutamic acid (d/l-ratio) in the produced PGA was 5/95. In contrast, d/l-ratio of produced PGA by the transformants introduced with *pgsBCA* genes (control strains) was 75/25. In conclusion, *B*. *subtilis* without *pgsA* gene could over-produce PGA with an l-rich enantiomeric ratio.

## Introduction

Poly-γ-glutamic acid (PGA) is a biopolymer comprising linear amide linkages of glutamate between α-amino and γ-carboxyl groups (Ashiuchi and Misono 2005). PGA is the predominant source of stickiness in the Japanese traditional food Natto, which is made by fermenting steamed soybeans using *Bacillus subtilis* var. natto (Saito et al. 1974). PGA has a wide range of applications, because it is edible, biocompatible, biodegradable, and can be produced from sustainable materials (Luo et al. 2016). Accordingly, PGA and its derivatives are used as food additives, cosmetic materials, health-care materials, drug carriers, flocculating agents, and soil conditioners (Buescher and Margaritis 2007; Luo et al. 2016). Thus, improved supply of low-cost PGA can expand the use of this bio-material.

Ashiuchi and Misono previously suggested that PGA production may be achieved using Gram-positive *Bacilli* such as *B. anthracis* and the extremely halophilic archaeon *Natrialba aegyptiaca*. These microbes produce PGA polymers of 10 to more than 1000 kDa (kilo Dalton) with specific d/l-ratios (Ashiuchi and Misono 2002). Specifically, d-PGA homopolymers comprise only d-glutamate, whereas l-PGA homopolymers comprise only l-glutamate, and dl-PGA copolymers contain both enantiomers of glutamate. Among these, homopolymers have strong resistance to phagocytosis (Makino et al. 1989) and can act as protein stabilizers (Yamasaki et al. 2010).

PGA is synthesized in *Bacillus* strains by an ATP-dependent transmembrane enzyme complex that catalyzes amide ligation (Ashiuchi et al. 2001). This complex comprises PgsBCA proteins in *B. subtilis* NBRC 3336 (Ashiuchi et al. 1999), YwsC and YwtAB in *B. subtilis* Marburg 168 (Urushibata et al. 2002), and CapBCA in *B. anthracis* TE702 (Makino et al. 1989). Previous studies also indicate that PgsB possesses glutamate-dependent ATPase activity, whereas PgsC has a hydrophobic structure in its transmembrane domain and facilitates PGA excretion (Ashiuchi et al. 2001). PgsA have been classified as A1-type anchor proteins and located on outer cell surface (Leenhouts et al. 1999; Narita et al. 2006).

In a previous study, Ashiuchi et al. reported that glutamate-dependent ATPase activities only in PgsBCA and PgsBC complexes, and showed that PgsBCA is about 3-fold more active than PgsBC (Ashiuchi et al. 2001). These investigators also suggested that PgsB and PgsC form a tight association, whereas only week interactions were observed between PgsBC and PgsA. Moreover, their experiments with d-xylose-inducible expression vectors showed no PGA production in the absence of the *pgsA* gene in mutant *B. subtilis* ISW1214 cells, suggesting that all components of the PgsBCA complex are essential for PGA production (Ashiuchi et al. 2006).

In a contrasting study, Urushibata et al. showed that purified histidine-tagged YwsC (homolog of PgsB) synthesized PGA alone in vitro system (Urushibata et al. 2002). In addition, PGA production was observed in the absence of YwtB (homolog of PgsA), although this was much less than in wild type cells. Hence, YwtB may be necessary for maximal PGA production.

Given the discrepancies between previous studies, it remains unclear which components of PgsBCA are required for efficient PGA production, and which could be exploited for high yield commercial PGA production. To address these issues, we investigated key components of the PGA synthetic machinery by constructing vectors, which assembled using a high copy-number plasmid vector and the σA-dependent promoter from the *egl*-*S237* gene (Hakamada et al. 2000). These molecular approaches reportedly achieved constitutive gene expression in *B. subtilis* during the log-growth phase (Manabe et al. 2011). Thus, we constructed mutant *Bacillus subtilis* strains by transforming with various combinations of *pgsBCA* in vectors that allowed high-level expression of genes. Following transformation by the *pgsBC* gene-expression vectors without *pgsA* gene, recombinant *B*. *subtilis* produced the highest yield of PGA, as reported previously (Luo et al. 2016), and the ensuing product had increased l-glutamic acid contents. These results demonstrate a procedure for over-production of the biopolymer l-PGA using recombinant *B. subtilis* having no *pgsA* gene.

## Materials and methods

### Bacterial strains and plasmids

*Bacillus subtilis* Marburg 168 was used as parental strain for construction of *pgs*-gene deletion mutants. The alkaliphilic laboratory strain *Bacillus* sp. KSM-S237 (FERM BP-7875) produces alkaline cellulase and was used as a gene resource for the promoter region of the *egl*-*S237*. The laboratory strain *Bacillus* sp. KSM-366 (FERM BP-6262) produces alkaline cellulase and PGA, and was used as a source of heterologous *pgs*-genes. *Bacillus amyloliquefaciens* NBRC 3022 (Hara et al. 1992) and *Bacillus licheniformis* ATCC 9945a (Potter et al. 2001) were also used as gene sources of PGA producers. *Oceanobacillus iheyensis* JCM 11309 (Takami et al. 2002) was used as a source of genes with low homology to those from *B*. *subtilis*. *Escherichia coli* HB101 and the plasmid pHY300PLK were purchased from Takara Bio Inc. (Kyoto, JPN). The plasmids pC194 (Ehrlich 1977) and pHYS237 (Manabe et al. 2011) were used as sources of the chloramphenicol-resistant gene (Cmr) and as templates for cloning of plasmid fragments with *egl*-*S237* promoter region.

### Bacterial growth media

PGA producing cells were pre-cultured in Luria–Bertani (LB) medium, and were then cultured in 2xL/Mal medium containing 2% (w/v) tryptone (Difco), 1% (w/v) yeast extract (Difco), 1% (w/v) NaCl, 7.5% (w/v) maltose monohydrate, and 7.5-ppm MnSO_4_·4–5H_2_O or in 2xL/Mal+E8 medium, which was supplemented with 8% (w/v) mono-sodium glutamate monohydrate. All media were supplemented with 15-ppm tetracycline for culture of transformants. *B*. *subtilis* and *E*. *coli* strains were transformed as described previously (Manabe et al. 2011). *B*. *subtilis* transformants were selected on LB agar plates containing 10-ppm chloramphenicol (LBCm), and were regenerated on DM3 media containing 30-ppm tetracycline (DM3Tc) (Chang and Cohen 1979). *E*. *coli* transformants were selected on LB agar plates supplemented with 15-ppm tetracycline (LBTc). All components of culture media were purchased from Wako Pure Chemicals (Osaka, JPN). Antibiotics for selection media were purchased from Sigma-Aldrich (St. Louis, MO, USA).

### General DNA manipulations

Restriction enzymes, DNA ligase, and DNA polymerase were purchased from Takara Bio Inc. (Kyoto, JPN). Expression vectors for heterologous *pgs*-genes were constructed using In-Fusion Cloning Kits (Takara Bio USA, Inc., California, USA). Genomic DNA was prepared according to previously described procedures (Saito and Miura 1963). PCR products and plasmid samples were purified using High Pure PCR Product Purification Kits and High Pure Plasmid Isolation Kits (Roche diagnostics, Basel, CHE). DNA sequences were analyzed using the Big-Dye Terminator v3.1 Cycle Sequencing Kits (Thermo Fisher Scientific, Massachusetts, USA) and Applied Biosystems 3130xl Genetic Analyzers (Thermo Fisher Scientific, Massachusetts, USA). DNA and protein sequences were searched using GENETYX program ver. 13 (GENETYX, Co., Tokyo, JPN).

### Mutant strain construction

To construct *B*. *subtilis* mutant ∆3, DNA fragments for deletion of *pgsBCA* were amplified using PCR with the primer pairs of BCA-Dw Fw and BCA-Dw Rv (BCA-Dw fragment), Cmr Fw and Cmr Rv (Cmr fragment), and BCA-Up Fw and BCA-Up Rv (BCA-Up fragment). The resulting fragments contained overlapping sequences as shown in Table 1, and were ligated using the spliced overlapping-extension (SOE)-PCR method (Horton et al. 1989) with the primer pair of BCA-Dw Fw and BCA-Up Rv. Competent *B*. *subtilis* cells were exposed to the resulting ligated fragment (approximately 2.9 kb) using the Spizizen protocol (Young and Spizizen 1963) and the transformants were selected on LBCm. Gene replacement efficiency was confirmed using direct colony PCR with the primer pair of BCA-Dw Fw and BCA-Up Rv. The size of the PCR product from targeted transformants was about 2.1 kb, which was shorter than those in wild type strains (about 5.0 kb).

**Table 1:** Primers used for construction of mutants and plasmids

Primer names	Sequence (5′→3′)
Cmr Fw	CAACTAAAGCACCCATTAGTTCAACAAACG
Cmr Rv	CTTCAACTAACGGGGCAGGTTAGTGAC
BCA-Dw Fw	CAAGCCCCGAGCAATCA
BCA-Dw Rv	CTAATGGGTGCTTTAGTTGTCGGAGTGATAAAGATGAAATTTGTC
BCA-Up Fw	CTGCCCCGTTAGTTGAAGTGCTTTTCGACATCTCCTT
BCA-Up Rv	AAGGGTTTGTGATATCCGG
P_S237 Fw	CAACTAAAGCACCCATTAG*GGATCC*AACAGGCTTATATTTAGAG
P_S237 Rv	CATCATATTACCTCCTAAATATTTTTAAAGTA
Bsu pgsA Rv	CG*AAGCTT*AGATGGCTTTGACAAATTTCATC
Bsu pgsC Rv	CCC*AAGCTT*GACCTTCGGCGTTTCCGCT
Bsu pgsB Rv	CCC*AAGCTT*GGCAGCGAATTTTCTGCGTCC
Bsu pgsA Fw	ATTTAGGAGGTAATATG**ATG**AAAAAAGAACTGAGCTTTCATG
Bsu pgsC Fw	ATTTAGGAGGTAATATG**ATG**TTCGGATCAGATTTATACATC
Bsu pgsB Fw	ATTTAGGAGGTAATATG**ATG**TGGTTACTCATTATAGCCTG
Bsu pgsBA Fw	CGTAAGCTAGGGGGAAATGCAGACGATGAAAAAAGAACTGAGCTTTCATG
pHY-P_S237 Rv	**CAT**CATATTACCTCCTAAATATTTTTAAAGTA
pHY Fw	TAG*AAGCTT*GGGCAAAGCGTTTTTCCA
Bam pgsB Fw	GGAGGTAATATG**ATG**TGGTTACTCATTATAGCCTGTG
Bli pgsB Fw	GGAGGTAATATG**ATG**TGGGTAATGCTATTAGCCTGTG
Obi pgsB Fw	GGAGGTAATATG**ATG**CTAGAAGAATTTTTAATCATTT
Bam pgsC Rv	TTGCCC*AAGCTT*CTACCTTCCCTGTCAGGATGCAG
Bli pgsC Rv	TTGCCC*AAGCTT*CTACTTCCGACCCAAGTAAAGAC

Italic sequences represent synthesized restriction enzyme sites; underlined sequences represent homologous regions for spliced overlapping extension (SOE)-PCR; bold sequences represent initiation codons for the open reading frames (ORFs) of each gene

### Plasmids construction

**Fig. 1:**
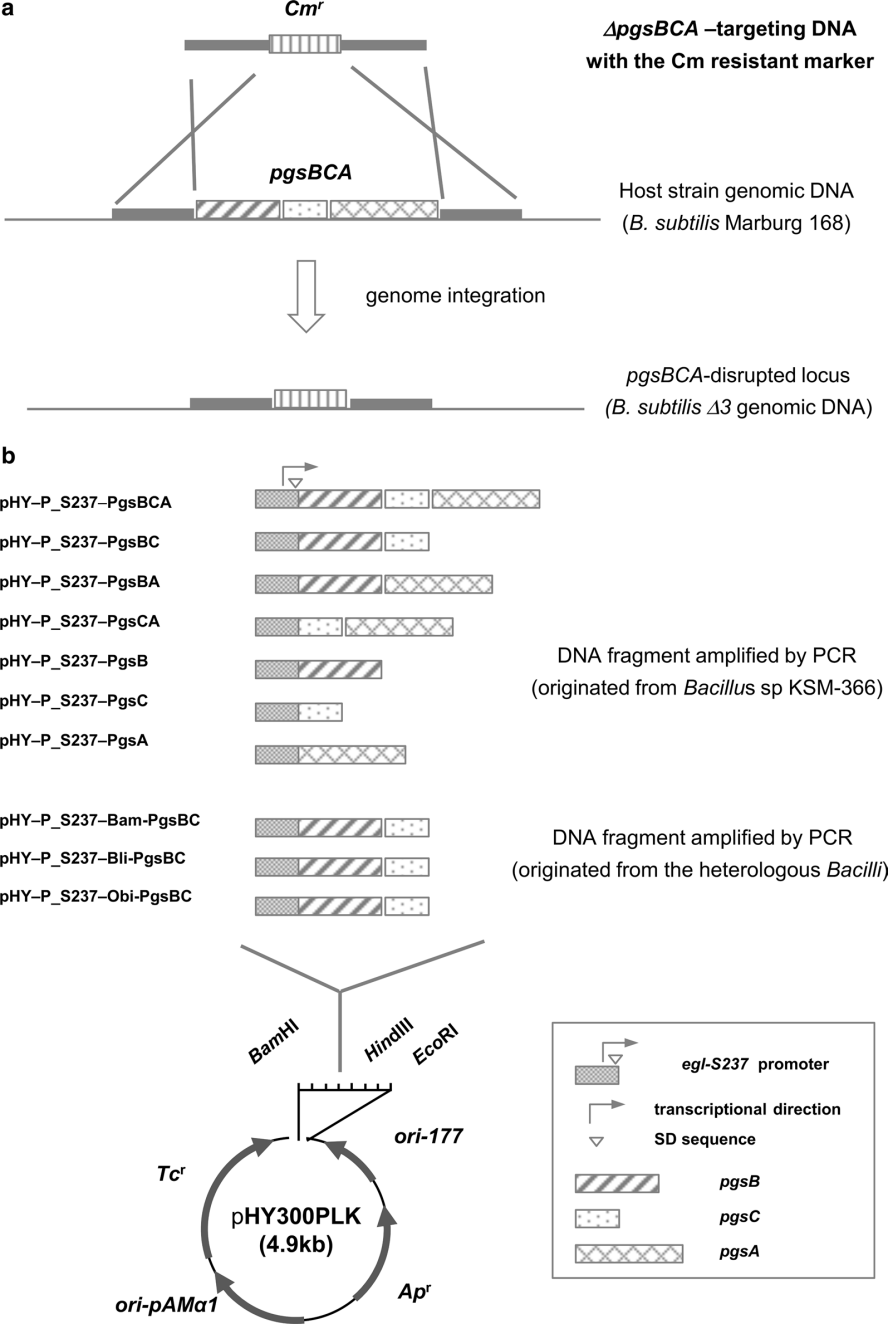
Construction of *pgsBCA* deletion mutants and *pgs*-gene expression vectors. **a** Deletion of *pgsBCA* genes from *B. subtilis* Marburg No.168 by double crossover homologous recombination. **b** Construction of *pgs*-gene expression vectors. DNA fragments (*pgsBCA*–*pgsA*) were amplified using spliced overlapping-extension (SOE)-PCR and were cloned into the plasmid pHY300PLK using restriction enzyme digestion. The heterologous *Bacillus pgsBC* fragments Bam-pgsBC, Bli-pgsBC, and Obi-pgsBC were then cloned using an in-fusion recombination cloning system

To construct plasmids for *pgs*-gene expression, the promoter region of the *egl*-*S237* was amplified using PCR with genomic DNA from *Bacillus* sp. KSM-S237 as a template and the primer pair of P_S237 Fw and P_S237 Rv, and the amplified fragment was named P_S237. Similarly, *pgs*-gene fragments were PCR amplified using genomic DNA from *Bacillus* sp. KSM-366 and the primer pairs of Bsu pgsB Fw and Bsu pgsA Rv (PgsBCA), Bsu pgsB Fw and Bsu pgsC Rv (PgsBC), Bsu pgsC Fw and Bsu pgsA Rv (PgsCA), Bsu pgsB Fw and Bsu pgsB Rv (PgsB), Bsu pgsC Fw and Bsu pgsC Rv (PgsC), and Bsu pgsA Fw and Bsu pgsA Rv (PgsA). Subsequently, the fragments P_S237 and PgsBCA, PgsBC, PgsCA, PgsB, PgsC and PgsA were ligated using SOE-PCR to produce P_S237–PgsBCA, P_S237–PgsBC, P_S237–PgsB–PgsA, P_S237–PgsCA, P_S237–PgsB, P_S237–PgsC, and P_S237–PgsA. These ligated fragments were then digested with *Bam*HI and *Hin*dIII and were introduced into the multi-cloning site of pHY300PLK. The resulting plasmids were named pHY–P_S237–PgsBCA, pHY–P_S237–PgsBC, pHY–P_S237–PgsBA, pHY–P_S237–PgsCA, pHY–P_S237–PgsB, pHY–P_S237–PgsC, and pHY–P_S237–PgsA and were used as expression vectors for *pgs*-genes (Fig. 1).

### Cloning of homologous *pgs*-genes

DNA fragments from the plasmid pHYS237, which digested with *Xba*I at multiple cloning sites of vector and open reading frame (ORF) of the *egl*-*S237*, were prepared as templates for PCR. DNA fragments as plasmid vector carrying the promoter region of *egl*-*S237* were amplified using PCR with the primer pair of pHY-P_S237 Rv and pHY Fw. And then, the homologous *pgsBC* of *B*. *subtilis* were also cloned using PCR. The *pgsBC* of *B*. *amyloliquefaciens* NBRC 3022 was amplified using PCR with genomic DNA as template and the primer pair of Bam pgsB Fw and Bam pgsC Rv, and the resulting fragment was named Bam-PgsBC. Similarly, the *pgsBC* fragment from *B*. *licheniformis* ATCC 9945a was prepared using the primer pair of Bli pgsB Fw and Bli pgsC Rv (Bli-PgsBC), and the *pgsBC* fragment from *O*. *iheyensis* JCM 11309 was prepared using the primer pair of Obi pgsB Fw and Obi pgsC Rv (Obi-PgsBC). Subsequently, directional cloning was performed using In-Fusion Cloning Kits with the DNA fragments pHY–P_S237 and Bam-PgsBC, pHY–P_S237 and Bli-PgsBC, and pHY–P_S237 and Obi-PgsBC. The constructed plasmids pHY–P_S237–Bam-PgsBC, pHY–P_S237–Bli-PgsBC, and pHY–P_S237–Obi-PgsBC were used as the expression vectors for heterologous *pgs*-genes.

### PGA production of transformants

Expression vectors of *pgsBC*-genes and the control vector pHY300PLK were introduced using the protoplast transformation method (Chang and Cohen 1979). And then, the resulting transformants were selected on DM3Tc as single colonies, and were inoculated into media using an inoculating-loop. PGA production and cell growth were assessed as end-points using ϕ 25-mm culture tubes (NICHIDEN RIKA-GLASS Co., Ltd., Kobe, JPN) containing 5 mL of 2xL/Mal and/or 2xL/Mal+E8 medium. Culture tubes were shaken using a reciprocal shaker HR-1000R (PRECi Co., Ltd., Tokyo, JPN) at 250 rpm and 37 °C for 72 h. In addition, time course experiments using 500-mL flask with baffles (Biott, Co., Tokyo, JPN) were examined, transformants on DM3Tc were pre-cultured in ϕ 25-mm culture tubes containing 5 mL of LB supplemented with 15-ppm tetracycline. After reciprocal shaking at 250 rpm (30 °C) for 20 h as pre-cultures, these broths were inoculated to an optical density at 600 nm (OD_600_) of 0.1 in main cultures using shake-flasks containing 30 mL of 2xL/Mal and/or 2xL/Mal+E8 medium. Subsequently, these flasks were shaken using a rotary shaker (TB-98R; PRECi Co., Ltd.) at 210 rpm and 37 °C for 72 h. During this cultivation period, cell growth was periodically monitored by measuring OD_600_ using a UV-2800A spectrophotometer (Hitachi High-technologies Co, Tokyo, JPN). If necessary, samples were diluted with 1% (w/w) NaCl solution prior to determining OD_600_. Serial culture broths were also centrifuged at 14,000×*g* for 30 min at room temperature, and supernatants were then analyzed for PGA production using HPLC.

### PGA analyses

PGA contents and molecular weights were determined using size exclusion chromatography. All samples were diluted with HPLC eluent, and were then filtered through 0.45-μm Durapore membranes (Multiscreen MSHVN4510; Merck Millipore, Massachusetts, USA). Samples were then analyzed using HPLC with a tandem-jointed SEC column (TSKgel G6000PWXL + G4000PWXL; TOHSO, Tokyo, JPN). Samples were eluted with 0.1-M Na_2_SO_4_ at a flow rate of 1.0 mL/min at 50 °C, and eluted peaks were detected using a UV detector at a wavelength of 210 nm. PGA contents were then calculated using a calibration curve that was prepared with food grade PGA additive (Meiji Food Materia, Tokyo, JPN), and the molecular weight of PGA was determined using the authentic pullulan STANDARD P-82 (Showa Denko, Tokyo, JPN). The stereo-chemical composition of PGA was determined as described previously (Ashiuchi et al. 2004; Ogawa et al. 1997). Produced PGA was then collected from culture broth using EtOH precipitation and was hydrolyzed with 6-N HCl at 105 °C for 15 h. Total glutamic acid contents of hydrolyzed samples were measured using a L-8900 amino acid analyzer (Hitachi High-technologies Co, Tokyo, JPN), and l-glutamic acid contents were determined using l-glutamic acid assay kits (Kikkoman, Chiba, JPN). d-glutamic acid contents were calculated as the difference between total and l-glutamic acid contents, and d/l-ratios of PGA were then calculated.

### Sequence alignment and structure prediction

Sequences were suitably aligned in multiple alignment analyses using the GENETYX program. Degrees of alignments were calculated using the “Multiple Alignment” program (Gap penalty; GAP Insert [− 12], GAP Extend [− 4]), and degrees of homology were assessed using the “Fasta Homology Search; fastp (protein–protein)” program. Secondary structure predictions were made using the SOSUI system (SOSUI engine ver. 1.11; http://harrier.nagahama-i-bio.ac.jp/sosui/) (Hirokawa et al. 1998).

### Nucleotide sequences and accession numbers

Sequencing analyses showed that the cloned *pgsB* from *Bacillus* sp. KSM-366 had an amino acid substitution (aspartate to glycine) at residue 27 (D27G) compared with the PgsB of *B*. *subtilis* Marburg 168 (BSORF ID, BG12531). Alternatively, the cloned *pgsC* from *Bacillus* sp. KSM-366 was identical to that of *B*. *subtilis* Marburg 168 (BSORF ID, BG12532). Further sequencing data of cloned *pgsBC* from *B*. *amyloliquefaciens* NBRC 3022 and *B*. *lichenifromis* ATCC 9945a gave similar results to those for *B*. *amyloliquefaciens* FZB42 (Genbank ID, ABS75634.1 and ABS75633.1) and *B*. *licheniformis* ATCC 14580 (GenBank ID, AAU25282.1 and AAU25281.1). Sequencing data for the cloned *pgsBC* of *Bacillus* sp. KSM-366, *B*. *amyloliquefaciens* NBRC 3022, and *B*. *licheniformis* ATCC 9945a were submitted to the DNA Data Bank of Japan (DDBJ; http://www.ddbj.nig.ac.jp). Sequencing data for each gene can be accessed using DDBJ accession numbers LC279207, LC279208, and LC279200.

## Results

### PGA production by *Bacillus* transformants

To identify genes that can be used for over-production of PGA in *B*. *subtilis*, we constructed seven *pgs*-gene expression vectors (Fig. 1b), and the transformed *B*. *subtilis ∆*3 strain with *pgs*-gene expression vectors using the protoplast method. Prepared transformants were cultivated in culture tubes with shaking for 72 h at 37 °C in either 2xL/Mal medium or 2xL/Mal+E8 medium. PGA production by transformants was estimated using high-performance liquid chromatography (HPLC).

As shown in Table 2, both transformants with pHY–P_S237–pgsBCA (P_BCA/*∆*3) and pHY–P_S237–pgsBC (P_BC/*∆*3) produced and secreted PGA into 2xL/Mal+E8 medium, with yields of 3.0 and 26.0 g/L, respectively. The P_BC/*∆*3 strain also secreted PGA into 2xL/Mal medium with a final PGA yield of 4.0 g/L, whereas transformants harboring the *pgs*-gene expression vectors pHY–P_S237–pgsBA, pHY–P_S237–pgsCA, pHY–P_S237–pgsB, pHY–P_S237–pgsC, pHY–P_S237–pgsA, and control vector pHY300PLK (P_ BA/*∆*3, P_CA/*∆*3, P_B/*∆*3, P_C/*∆*3, P_A/*∆*3, and P_300/*∆*3, respectively) did not produce PGA in 2xL/Mal medium with or without l-glutamate.

**Table 2:** Growth and PGA production by *Bacillus* transformants

Strain	2xL/Mal			2xL/Mal+E8		
	OD_600_	Mw/10^3^	Prod (g/L)	OD_600_	Mw/10^3^	Prod (g/L)
P_300/*∆*3	34 ± 3.3	nd	nd	47 ± 1.6	nd	nd
P_BCA/*∆*3	34 ± 6.2	nd	nd	40 ± 10	1000 ± 200	3.0 ± 0.7
P_BC/*∆*3	14 ± 4.1	400 ± 100	4.0 ± 0.3	16 ± 4.6	1100 ± 200	26.0 ± 3.1
P_BA/*∆*3	38 ± 2.7	nd	nd	30 ± 2.8	nd	nd
P_CA/*∆*3	38 ± 1.0	nd	nd	41 ± 3.2	nd	nd
P_B/*∆*3	33 ± 3.8	nd	nd	27 ± 2.4	nd	nd
P_C/*∆*3	34 ± 1.6	nd	nd	40 ± 2.3	nd	nd
P_A/*∆*3	37 ± 1.5	nd	nd	47 ± 2.3	nd	nd

Data are presented as means of five independent experiments ± SE (n = 5)

In further experiments, cell growth was evaluated according to OD_600_ of culture broth. As shown in Table 2, growth of the P_BC/*∆*3 strain was substantially lower than that of the control strain P_300/*∆*3 in both media types, whereas growth of the other transformants were similar to control strains.

### Time course of PGA production by transformants

**Fig. 2:**
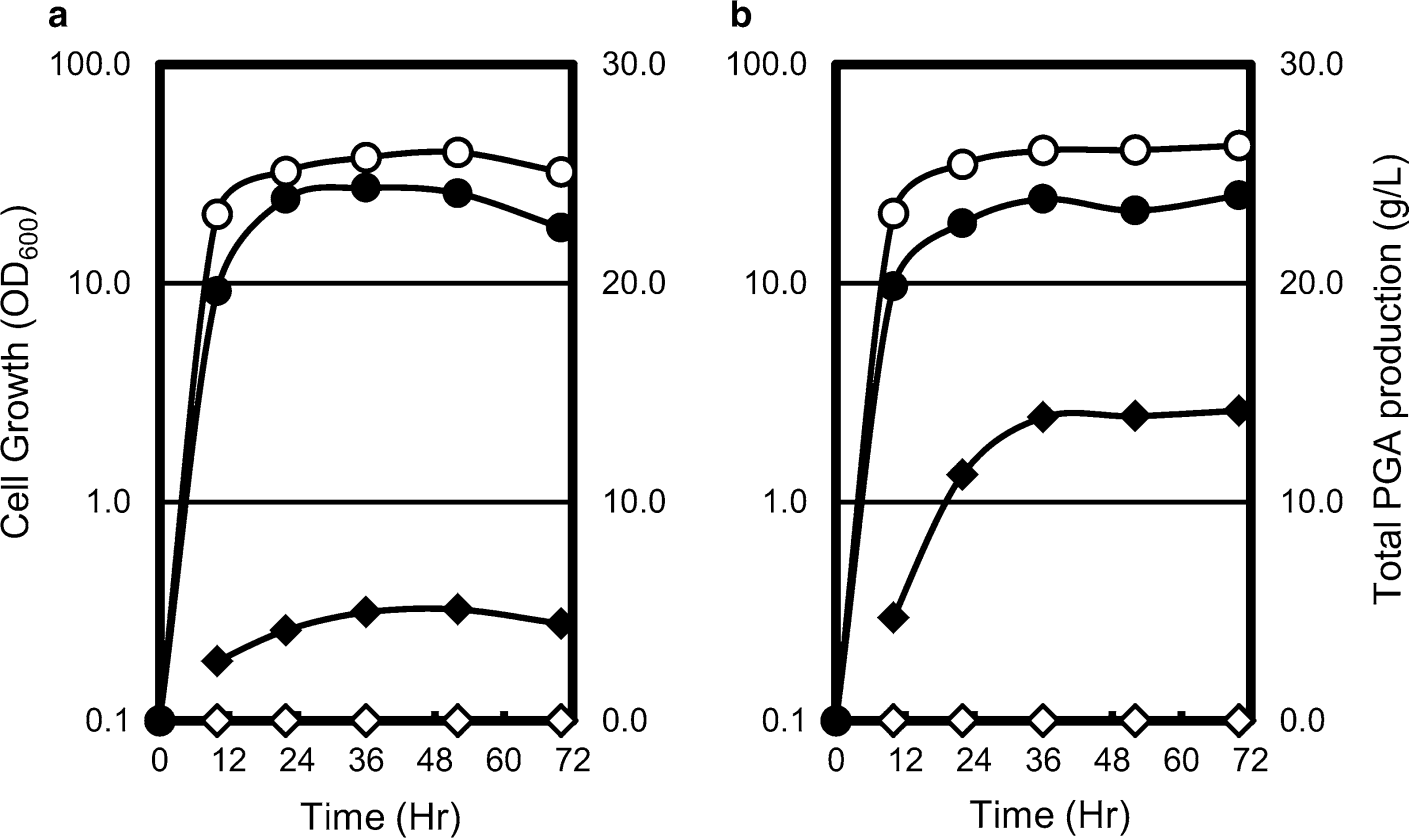
Time course of PGA production by **a** P_300/*∆*3 and P_BC/*∆*3 in 2xL/Mal medium and **b** P_300/*∆*3 and P_BC/*∆*3 in 2xL/Mal+E8 medium. Data are presented as means of five independent experiments. Symbols: opened circles, cell growth of P_300/***∆***3; opened diamonds, PGA production by P_300/***∆***3; closed circles, cell growth of P_BC/***∆***3; closed diamonds, PGA production by P_BC***∆***3

To further investigate PGA production and cell growth of the P_BC/∆3 strain, time course experiments were performed during batch fermentation in using 2xL/Mal medium or 2xL/Mal+E8 medium. As shown in Fig. 2, transformants exhibited triphasic growth curves in both media. Specifically, during the log-growth phase from 0 to 12 h, OD_600_ values increased exponentially from 0.1 to more than 10 in both media. Subsequently, a late log-growth phase was observed until the 36 h time point, with modest increases in OD_600_ values, and a stationary phase was observed thereafter with no increase in OD_600_. In comparisons of P_BC/*∆*3 and P_300/*∆*3 (control strain), cell growth of the P_BC/*∆*3 strain was decreased to 60–70% of the control strains in 2xL/Mal medium, and was decreased to 50–60% of the control strains in 2xL/Mal+E8 medium.

PGA production was detectable at 10 h after inoculation of cultures, and reached the maximum level at 36 h. Maximum PGA production reached 5 g/L (Mw/10^3^ = 2600) in 2xL/Mal medium and 14 g/L (Mw/10^3^ = 2100) in 2xL/Mal+E8 medium. Furthermore, PGA contents of culture broth were maintained at the maximum level after 36 h, and the molecular weights of PGA were maintained during the 72 h cultivation.

### Amino acid sequence alignment and structural estimation

**Fig. 3:**
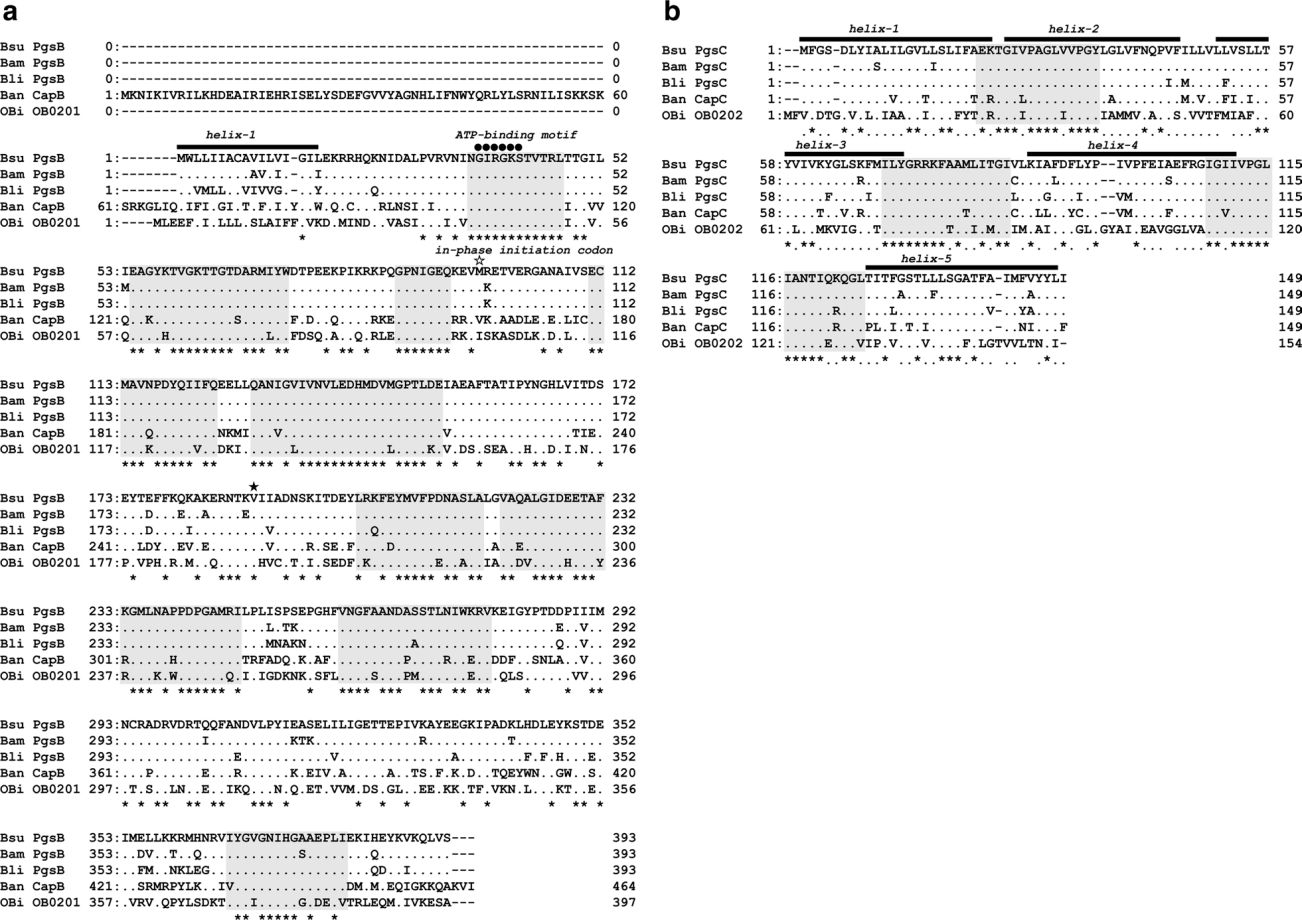
Multiple alignments of **a** PgsB and **b** PgsC proteins. Sequence alignments were performed using the GENETYX program. Identical residues are shown by periods (.) and sites with 100% homology are marked with asterisks (*) on the bottom of the line. Predicted consensus sequences are shaded in Gray tones. Secondary structure prediction was performed using the SOSUI program. Predicted transmembrane helix structures are shown as black lines on the tops of lines. Symbols: closed circles, ATP-binding motif; opened star, the initiation codon of in-phase overlapping *pgsB* genes of *B. subtilis*; closed star, the initiation codon of in-phase overlapping *capB* genes of *B. anthracis*

To characterize the PGA synthetase complex, we performed amino acid sequence alignments using the GENETYX program and determined homology between PgsBC proteins. As shown in Fig. 3, all of the present PgsB had the conserved sequence GIRGKS (residues 37–42 of Bsu-PgsB), which was presumed to be the ATP-binding motif (Aboulmagd et al. 2000; Ashiuchi et al. 2001). Furthermore, the in-phase initiation codon (Urushibata et al. 2002) of the overlapping gene (97Met of Bsu-PgsB) was conserved in present proteins but not in Ban-PgsB and Obi-PgsB.

Sequence homology was determined between PgsB from *B*. *subtilis* Marburg 168 (Bsu) and the other heterologous PgsB, and matching scores were 92% for PgsB from *B. amyloliquefaciens* NBRC 3022 (Bam-PgsB), 89% for *B. licheniformis* ATCC 9945a (Bli-PgsB), 67% for *B. anthracis* TE702 (Ban-PgsB), and 55% for *Oceanobacillus iheyensis* JCM 11309 (Obi-PgsB). Similarly, homology searches for PgsC gave matching scores of 93% for the PgsC from *B. amyloliquefaciens* NBRC 3022 (Bam-PgsC), 89% for *B. licheniformis* ATCC 9945a (Bli-PgsC), 77% for *B. anthracis* TE702 (Ban-PgsC), and 51% for *Oceanobacillus iheyensis* JCM 11309 (Obi-PgsC).

In structure estimations of PgsB and PgsC using the SOSUI program (ver. 1.11) as shown in Fig. 3, all PgsB except Bli-PgsB had hydrophobic moieties (residues 1–23 of Bsu-PgsB) in N-terminal regions, which comprise transmembrane helixes. The structure of PgsC was also predicted using the SOSUI program, which indicated the presence of five transmembrane helices (residues 1–23, 25–47, 51–72, 77–99, and 125–147 of Bsu-PgsC), one intracellular loop (residues 73–87 of Bsu-PgsC), and one extracellular loop (residues 111–125 of Bsu-PgsC).

### PGA production by transformants harboring heterologous *pgsBC* genes

To investigate the potential for over-production of PGA in *B. subtilis* expressing heterologous PgsBC, *B*. *subtilis* ∆3 strains were transformed with a series of heterologous *pgs*-gene expression vectors (pHY–P_S237–Bam-pgsBC, pHY–P_S237–Bli-pgsBC, and pHY–P_S237–Obi-pgsBC; Fig. 1a). The prepared transformants were then cultivated in shaking flasks containing 2xL/Mal+E8 medium for 72 h at 37 °C. The transformants harboring pHY–P_S237–Bam-pgsBC (P_Bam-BC/*∆*3), pHY–P_S237–Bli-pgsBC (P_Bli-BC/*∆*3), and pHY–P_S237–Obi-pgsBC (P_Obi-BC/*∆*3) produced 3.3-, 1.4-, and 0.7-g PGA/L at molecular weights of 1100 k, 2700 k, and 3300 k, respectively.

### Identification of stereo-chemical compositions

To assess d/l-ratios of glutamate in produced PGA, polymers were isolated from culture broth samples and were acid-hydrolyzed as described above. As shown in Table 3, the d/l-ratio of PGA from P_BCA/∆3 transformants, which harbored all components of the PgsBCA complex, was 75/25, and this value is close to that of PGA in the Japanese traditional food Natto (Ashiuchi 2013; Nagai et al. 1997). In contrast, the d/l-ratio of PGA from the P_BC/*∆*3 strain was 4/96, indicating a marked increase in l-glutamic acid. Furthermore, PGA from P_Bam-BC/*∆*3, P_Bli-BC/*∆*3, and P_Obi-BC/*∆*3 transformants had d/l-ratios of 4/96, 2/98, and 11/89, respectively.

**Table 3:** Chirality of PGA from transformants

Strain	Medium^a^	OD_600_	Mw/10^3^	Prod (g/L)	d/l-ratio
P_BC/Δ3	l-Glu	29.6	2000	35.3	4/96
P_BC/Δ3	d-Glu	17.5	600	3.1	4/96
P_Bam-BC/Δ3	l-Glu	37.9	1100	2.5	4/96
P_Bli-BC/Δ3	l-Glu	36.4	2800	1.4	2/98
P_Obi-BC/Δ3	l-Glu	39.0	1900	0.7^b^	11/89

^a^Medium; l-Glu was used 2xL/Mal+E8 (which contained 0.43M l-glutamic acid) and d-Glu was used 2xL/Mal, containing 0.43M d-glutamic acid and adjusted to the same pH as 2xL/Mal+E8 with NaOH. Data are presented as means of five independent experiments

^b^PGA production of P_Obi-BC/Δ3 strains is representative of three-fifth or two-fifth of positive transformants (it seems to be unstable)

## Discussion

PGA is synthesized and secreted by several *Bacillus* strains (Ashiuchi 2013; Luo et al. 2016). In previous studies, various experimental maneuvers were applied to elucidate PGA synthesis. Makino et al. first reported that the CapBCA of *B*. *anthracis*, which are membrane-associated proteins, catalyzes the synthesis of D-PGA (Makino et al. 1989). Subsequently, Ashiuchi et al. cloned *B*. *subtilis* NBRC 3336 *pgsBCA*, which are homologous to the *capBCA* of *B*. *anthracis*, were characterized these genes as the membrane-associated PGA synthetases (Ashiuchi et al. 1999, 2001). In these studies, they concluded that all *pgsBCA* are essential genes for PGA synthesis. On the other hand, Urushibata et al. cloned the *ywsC* (homolog of *pgsB*) of *B*. *subtilis* NBRC 16449 (Urushibata et al. 2002), and demonstrated that the histidine-tagged YwsC solely synthesized PGA. They concluded that both YwsC and YwtA (homolog of PgsC) are essential for PGA synthesis, and that YwtB (homolog of PgsA) seems to be required for maximum PGA production.

To identify the components responsible for over-production of PGA, we used the vectors that allowed high-level expression of genes and the *B. subtilis* mutant strain (*∆*3). Our experimental data indicated that the transformants P_BCA/*∆*3 and P_BC/*∆*3 produced PGA, indicating that PgsA is not essential for PGA production. In addition, transformants harboring either pHY–P_S237–PgsCA or pHY–P_S237–PgsC were unable to produce PGA, indicating that PgsB is essential for PGA production. Furthermore, transformants with the vectors pHY–P_S237–PgsBA or pHY–P_S237–PgsB did not lead to PGA production, further confirming the requirement of PgsC for PGA production. In our present studies, *B*. *subtilis* having no *pgsA* produced abundant PGA and the produced PGA exhibited an l-rich enantiomeric ratio. These data are partially in agreement with those reported by Ashiuchi et al. who indicated glutamate-dependent ATPase activity of both PgsBCA and PgsBC (Ashiuchi et al. 2001). On the other hands, our findings contradict those showed by Ashiuchi et al. who indicated the inability of PGA production using *B*. *subtilis* MA41 strains (genomic *pgsBCA*-gene disruptants) and pWPGBC1, which d-xylose inducible gene expression vector for *pgsBC* (Ashiuchi et al. 2006). We speculated that there are differences between the substance-inducible promoter and the constitutive high-level expression promoter. Additionally, our findings were not consistent with the results by Urushibata et al. who exhibited that the purified histidine-tagged PgsB synthesized PGA from l-glutamate in vitro system (Urushibata et al. 2002). In the view of these experimental facts, there was a possibility that the high sensitivity analysis using radioactive-labeled substances detected PGA synthesis by only PgsB. From the viewpoint of nutrient metabolism, PGA production seemed to negatively influence over host cell-growth. Actually, the maximum OD_600_ value of the P_PgsBC/*∆*3 strain drastically decreased to that of control strains. In addition, the yield of PGA of P_PgsBC/*∆*3 strain in 2xL/Mal medium was higher than the initial glutamic acid content. These data indicated that other amino acids in the growth medium were utilized for PGA synthesis, and cell growth was reduced as a consequent. Moreover the P_PgsBCA/*∆*3 strains did not produce PGA in batch fermentation with shaking flasks (data not shown). These results suggested that over-expression of *pgsBCA* conceivably caused in stress like nitrogen starvation as d-glutamate auxotrophy (Ashiuchi et al. 2007). Also, as previously reported, PGA production was improved by NADPH regeneration in *B*. *licheniformis* WX-02 (Cai et al. 2017). Taken together, these data warrant speculation that PGA synthesis in vivo system seems like to be influenced by not only synthesis machinery but also nitrogen and energy metabolism.

In this study, we confirmed PGA production by the transformants harboring heterologous PgsBC. And then, these sequence homology and structure were verified. Although the homology was low between Bsu-PgsB and Obi-PgsB (55%) and between Bsu-PgsC and Obi-PgsC (51%), transformants harboring Obi-PgsBC produced PGA. Accordingly, structural estimations using the SOSUI program indicated that PgsB homologs all have one transmembrane helix, whereas PgsC homologs have five transmembrane helices. Moreover, predicted consensus regions were found in both PgsB and PgsC, and two conserved regions of PgsC were located in the loop structure. These results indicate that similarities in protein structures are more important than similarities in amino acid sequences. Similar interpretations were suggested in the ADP-forming amide bond ligase superfamily (Sheng et al. 2000; Urushibata et al. 2002). Assessments of d/l-ratios of produced PGAs showed that P_BCA/*∆*3 produced dl-PGA, whereas P_BC/*∆*3 produced l-PGA. Hence, l-PGA can be synthesized in the absence of PgsA, suggesting interactions between PgsA and racemase and/or conformational changes induced by binding of PgsA to PgsBC complex (Sheng et al. 2000).

In a previous study, Urushibata et al. showed that YwtA (homolog of PgsC) is a hydrophobic cell membrane protein that transports PGA in *B*. *subtilis* (Urushibata et al. 2002). Ashiuchi et al. suggested that PgsB and PgsC are naturally associated with the cell membrane and have glutamate-dependent ATPase activity (Ashiuchi et al. 2001). Our present data using growing cells suggest that the PgsBC complex is critical for excretion of excess PGA and that this over-production system causes stress to host cells. Thus, to further improve PGA production, studies will be designed to characterize interactions of PgsBC with cell membranes, and to resolve the competition between PGA production and cell growth so that glutamate, ATP, and NADPH can be optimally provided.

In conclusion, the present study shows that PgsBC is essential for over-production of PGA. Additionally, we developed a promising method for modifying d/l-ratios of PGA in favor of the useful material l-PGA (Yamasaki et al. 2010).

## Abbreviations

*PGA*: poly-gamma-glutamic acid
*D/L*-*ratio*: enantiomeric ratio of d- and l-glutamic acid
*kDa*: kilo Dalton
*Cmr*: chloramphenicol-resistant gene
*LB*: Luria–Bertani
*LBCm*: LB agar plates containing 10-ppm chloramphenicol
*DM3Tc*: DM3 media containing 30-ppm tetracycline
*LBTc*: LB agar plates containing 15-ppm tetracycline
*ORF*: open reading frame
*OD*_*600*_: optical density at 600 nm
